# Identifying the effects of scaffolding on learners’ temporal deployment of self-regulated learning operations during game-based learning using multimodal data

**DOI:** 10.3389/fpsyg.2023.1280566

**Published:** 2023-11-09

**Authors:** Daryn A. Dever, Megan D. Wiedbusch, Sarah M. Romero, Kevin Smith, Milouni Patel, Nathan Sonnenfeld, James Lester, Roger Azevedo

**Affiliations:** ^1^School of Modeling, Simulation, and Training, University of Central Florida, Orlando, FL, United States; ^2^Department of Psychology, University of Central Florida, Orlando, FL, United States; ^3^Department of Computer Science, North Carolina State University, Raleigh, NC, United States

**Keywords:** self-regulated learning, game-based learning, scaffolding, multimodal data, transition probabilities

## Abstract

**Introduction:**

Self-regulated learning (SRL), or learners’ ability to monitor and change their own cognitive, affective, metacognitive, and motivational processes, encompasses several operations that should be deployed during learning including Searching, Monitoring, Assembling, Rehearsing, and Translating (SMART). Scaffolds are needed within GBLEs to both increase learning outcomes and promote the accurate and efficient use of SRL SMART operations. This study aims to examine how restricted agency (i.e., control over one’s actions) can be used to scaffold learners’ SMART operations as they learn about microbiology with Crystal Island, a game-based learning environment.

**Methods:**

Undergraduate students (*N* = 94) were randomly assigned to one of two conditions: (1) Full Agency, where participants were able to make their own decisions about which actions they could take; and (2) Partial Agency, where participants were required to follow a pre-defined path that dictated the order in which buildings were visited, restricting one’s control. As participants played Crystal Island, participants’ multimodal data (i.e., log files, eye tracking) were collected to identify instances where participants deployed SMART operations.

**Results:**

Results from this study support restricted agency as a successful scaffold of both learning outcomes and SRL SMART operations, where learners who were scaffolded demonstrated more efficient and accurate use of SMART operations.

**Discussion:**

This study provides implications for future scaffolds to better support SRL SMART operations during learning and discussions for future directions for future studies scaffolding SRL during game-based learning.

## 1. Introduction

In 2019, the US National Assessment of Educational Progress (NAEP) released a report that shows decreasing proficiency in science achievement levels as grade levels increase, where 24% of students in Grade 4, 32% of students in Grade 8, and 40% of students in Grade 12 demonstrated below basic proficiency. Within the same report, 50% of students in Grade 12 stated that they never or only once in a while engaged in scientific inquiry-related classroom activities [National Center for Education Statistics (NCES), 2019]. This report illuminates the decreasing scientific competency of students and provides researchers an avenue to understand how advanced learning technologies, such as game-based learning environments (GBLEs), can be integrated with traditional classroom instruction to increase learning outcomes. However, in a report about the uses of educational technology for instruction, only 33% of educators reported that they strongly agreed that technology within the classroom has helped their students become self-regulated learners (Gray and Lewis, 2021). Scaffolding techniques have been embedded within GBLEs to aid learners in their acquisition of domain knowledge (Chen and Law, 2016; Dever et al., 2020; Saleh et al., 2020; Taub et al., 2020; Kim et al., 2021; Chen et al., 2023), but few learning environments incorporate scaffolding techniques that are intended to support individual learners’ self-regulated learning (SRL) processes. As such, this study explores how scaffolding within a GBLE supports learners’ SRL processes and learning outcomes. This paper captures learners’ multimodal data (i.e., eye tracking, log files) to determine how learners temporally transition between SRL operations depending on the level of scaffolding provided and how these transitions are related to their learning outcomes. The findings from this study will be used to inform adaptive, individualized scaffolding within GBLEs to support learners in deploying SRL processes and increasing learning outcomes.

## 2. Scaffolding self-regulated learning during game-based learning

Self-regulated learning (SRL) is learners’ ability to monitor and change their own cognitive, affective, metacognitive, and motivational processes to achieve a goal (Winne and Azevedo, 2022). SRL has been touted as an attribute of a successful learner (Samruayruen et al., 2013; Karlen et al., 2020) as SRL is required to set goals, develop plans to reach those goals, deploy strategies during learning, reflect on learners’ progress toward goals and the effectiveness of deployed strategies, and re-prioritize or modify goals and plans to achieve greater learning outcomes (Winne, 2018). SRL is a complex process to deploy, especially while learning about a difficult subject (e.g., STEM topics), as it involves learners constantly monitoring and changing their SRL processes during learning (Azevedo et al., 2022; Dever et al., 2022). Deploying accurate and effective SRL strategies becomes even more challenging during game-based learning. This is due to the open-ended nature of GBLEs in which learners are able to explore the elements and affordances of the GBLE but are often left unsupported as most games for learning do not employ scaffolds that help learners engage in SRL. This section reviews how previous literature has captured and scaffolded SRL during game-based learning.

### 2.1. Capturing and examining self-regulated learning during game-based learning

SRL operations during learning with a GBLE can be difficult to capture using unimodal traditional methods such as click stream data and self-reports. Multimodal data affords researchers the opportunity to use multiple data streams to reveal learners’ internal SRL processes including the use of strategies as they learn with GBLEs (Azevedo et al., 2018, 2019; Alonso-Fernández et al., 2019; Di Mitri et al., 2019; Sharma and Giannakos, 2020; Giannakos et al., 2022). Multimodal data includes both subjective (e.g., self-report measures) and objective (e.g., log files, eye tracking) data channels that can capture physiological, verbal, behavioral, and contextual data during learning to reveal *how* learners interact with information, *what* SRL strategies learners may deploy, and *why* learners enact certain behaviors (Järvelä et al., 2019, 2021; Azevedo et al., 2022; Molenaar et al., 2023). In this article, we focus on utilizing both log-file and eye-tracking behavioral data to capture how learners engage in SRL during learning with a GBLE.

Log-file data captures the click streams, keystrokes, navigation, and other behaviors (e.g., opening/closing an interface window, selecting specific choices within an interface menu) within an open-ended environment such as a GBLE. These data report the actions and behaviors a learner takes as they interact with the system (Lim et al., 2021; Molenaar et al., 2023). Eye-tracking data, like log files, captures learning behaviors through learners’ eye gaze movements. The position of learners’ eye gaze in relation to the instructional materials presented in a learning environment can indicate attention allocation, reading behaviors, cognitive load of a task, problem-solving events, and contextualize decision-making processes (Chen and Tsai, 2015; Dever and Azevedo, 2019; Molenaar et al., 2023).

Previous studies have shown that multimodal data can be used to capture SRL behaviors as learners interact with a GBLE. Taub et al. (2017) used both of these process data to identify when learners engaged in both cognitive and metacognitive SRL strategy use while learning with a GBLE. Relating these behaviors to performance data, their findings found that using both eye-gaze and log-file data can capture the quality of learners’ SRL behaviors. Another study by Dever et al. (2020) used both data modalities to capture when learners engaged with instructional materials while learning with a GBLE. Results from this study showed that the use of both data modalities are essential for capturing when learners initiate specific SRL strategies, such as content evaluations. From these studies, it is critical to use multimodal data to capture SRL operations as learners interact with GBLEs to accurately and fully capture how learners deploy SRL operations during the learning process.

### 2.2. Scaffolds for self-regulated learning

Past literature has attributed successful learning to SRL in which learning gains increase when learners can identify the objectives of the task, set goals and plans to achieve the goals of the task, deploy SRL strategies that are effective in achieving those goals, and reflecting on their progress toward goals to constantly modify SRL behaviors that are more efficient and effective in successfully completing a task (Samruayruen et al., 2013; Dever et al., 2020; Karlen et al., 2020; Winne and Azevedo, 2022). Because of this, learners’ accurate and consistent use of SRL strategies during learning are essential for successful learning. As noted above, and as supported by prior literature (Azevedo et al., 2004, 2010; Taub et al., 2020; Cloude et al., 2021; Dever et al., 2023), SRL is challenging for learners to engage in, especially as instructional content and tasks become more complex in terms of difficulty. SRL is especially difficult to deploy during game-based learning because GBLEs are typically open-ended, requiring learners to self-navigate the environment and deploy SRL to manage goals, monitor progress toward goals, and deploy SRL strategies and operations. Scaffolds are tools that can be used either directly or indirectly to support learning objectives and outcomes (Stahl and Bromme, 2009; Winne, 2018; Winne and Marzouk, 2019; Dever et al., 2022; Azevedo and Wiedbusch, 2023; Wiedbusch et al., 2023). Explicit scaffolds are more overt in supporting learners where, for example, GBLEs may interrupt learners during the task to prompt them to engage in metacognitive processes (Dever et al., 2021; Zheng et al., 2023). Some GBLEs may be more implicit in scaffolding SRL. For example, several studies (e.g., Sawyer et al., 2017) have used restricted agency as a scaffold to guide how learners interact with the learning environment. Limiting agency acts as an indirect scaffold, as the learner is unaware of any controls the educator permits, to provide guidance to engagement meaningful to the GBLE’s learning gains. In GBLEs, agency may be restricted to guiding learners throughout the environment, promote learners’ monitoring of their progress toward goals, encourage learners to engage with certain instructional materials in the environment, etc. (Dever et al., 2022). Restricted agency as a scaffold has been examined in the context of Crystal Island, a GBLE that focuses on teaching learners about microbiology (Rowe et al., 2011; Dever et al., 2022).

#### 2.2.1. Crystal Island: review of past literature

Crystal Island is a game-based learning environment (GBLE) developed to support students as they learn about microbiology and improve scientific reasoning (Rowe et al., 2011; Dever et al., 2022). Studies have used Crystal Island to examine narrative within game-based learning (Lester et al., 2014; Dickey, 2020), scientific reasoning (Cloude et al., 2020), and planning and reflection (Rowe and Lester, 2015; Cloude et al., 2021; Dever et al., 2021), often using multimodal data collection methodologies (Taub et al., 2017; Cloude et al., 2020; Dever et al., 2020; Emerson et al., 2020). The Crystal Island environment typically affords learners full agency, or control over one’s own actions (Bandura, 2001); however, a few studies on Crystal Island have examined restricting agency as a scaffold of learning (Taub et al., 2019; Cloude et al., 2020).

For example, Taub et al. (2019) examined how learning outcomes differed across varying levels of agency, including fully unrestricted gameplay (i.e., full agency), partially restricted gameplay (i.e., partial agency), and vicarious learning (i.e., no agency). Learners who had partially restricted gameplay were restricted to a “golden path” for exploring the island and were required to interact with all content material. Learners with no agency followed a vicarious learning paradigm in which they did not play Crystal Island at all, but rather watched an expert playthrough. The highest learning outcomes were associated with those afforded partial agency while those with full agency tended to focus on extraneous distractor information that was not relevant to the problem. Learners with no agency tended to become uninterested in Crystal Island and disengaged from the task. Another study by Taub et al. (2020) examined the role of agency on learning, emotions, and problem-solving behaviors. This study, similar to Taub et al. (2019), found that learners with partial agency had the greatest learning outcomes compared to learners in the high and no agency conditions. However, this study did find that learners in both the high and partial agency conditions demonstrated greater frustration, confusion, and joy. This further supports the previous work, suggesting that learners were affectively disengaging during the no agency condition as well as behaviorally and cognitively disengaging. That is, agency is associated with multiple facets and processes of self-regulation including cognition, affect, and metacognition.

Dever et al. (2021) expanded prior work on agency in Crystal Island to examine the temporality of these differences. Specifically, they examined how learners engaged in information-gathering behaviors across instructional materials over time. Similar to the previous studies, Dever et al. (2021) found that learners who received partial agency had greater learning gains than learners who received full agency. Results from this study also showed that learners who had full agency had greater fixations on books and research articles over time and lower fixations on posters compared to learners with partial agency, contradicting the higher learning gains of the partial agency condition group. These findings show that learners who received differing levels of agency interact with GBLEs differently, indicating a need to fully understand how agency affects not just learners’ interactions with the environment but the relationship to learners’ deployment of SRL strategies. In other words, while prior studies have shown the limiting agency relates to how learners interact with instructional materials or experience affective states during learning, more research is needed to identify how learners engage in the process of SRL and how this is related to increased learning outcomes.

Despite previous work examining the relationship between agency and learning outcomes, there remains many questions about the relationship between agency and self-regulation. Specifically, questions on restricted agency as a scaffold supports learners’ temporal use of SRL processes. That is, how does agency, especially when deployed as a scaffold within open-ended GBLEs, impact self-regulatory processes? When do learners begin to shift from being gently guided by external regulators to maladaptively overlying on the support? How do learners move between various cognitive and metacognitive processes with and without these types of scaffolds? This study further examines these gaps by exploring how restricted agency scaffolds learners’ temporal transitions between theoretically defined cognitive and metacognitive processes as learners self-regulate within Crystal Island.

## 3. Theoretical framework: SRL SMART operations

The current study is grounded within Winne (2018) conditions, operations, products, evaluations, and standards (COPES) model of SRL. The COPES model describes how learners’ internal and external conditions influence how operations are deployed during learning which results in the products that evidence learning and how those products are evaluated against internal and external standards. Within the operations phase of COPES, five temporally-unfolding cognitive operations – searching, monitoring, assembling, rehearsing, and translating (SMART) – underly interactions between working memory and long-term memory during the operations phase of COPES. SMART processes are deployed to facilitate learners’ SRL strategy use: *searching* refers to the retrieval of goal-relevant information; *monitoring* is a cognitive process involving the comparison of information (i.e., products) against a standard*; assembling* involves the encoding of new information from the environment into working memory*; rehearsing* is a cognitive process which maintains information within working memory; and, *translating* involves searching for information in one modality and assembling that information in a different modality of representation (e.g., textual to graphical). These SMART operations are deployed by learners to generate new products (e.g., learning gains) as they learn within the GBLE.

These SMART operations may be behaviorally enacted within GBLEs. For example, *searching* can be defined as the movement within a GBLE as learners interact with instructional materials placed spatially throughout the environment. *Monitoring* operations can be identified as a learner completing performance measures, indicating a judgment of learning. *Assembling* operations can involve note-taking or summarizing where information is gathered from either single or multiple sources throughout the environment. *Rehearsing* operations include learners’ continual review of their new knowledge within working memory. Finally, *translating* operations include using one’s notes to make conclusions in which information from one or multiple sources is used within a new context. It is important to note that it is difficult to measure *rehearsing* directly through behavioral traces where most virtual learning environments and data capture methods (e.g., log-files) are unable to identify *when* and *for how long* learners rehearse information in working memory. While the SMART operations alone may not fully account for the range of functions performed by working memory relevant to learning, as addressed by other theoretical frameworks, these operations provide a foundation for understanding how learners regulate (i.e., monitor and control) their information processing to facilitate learning (Azevedo and Dever, 2022).

Learners’ SMART operations have been examined across several contexts. Zhang et al. (2022) examined how machine learning could detect when learners used SMART operations during learning on a mathematical platform. This study collected learners’ log-file data as well as verbalizations during mathematical problem-solving to construct a robust detector of when SMART operations were used by students. In another study, Hutt et al. (2021) examined how learners used SMART operations as they interacted with Betty’s Brain, a computer-based learning environment that maps learners’ understanding of causal scientific processes. This study used multimodal data, including interview, interaction, and survey data, and found that using multimodal data to automate detection of SMART operations is a reliable methodology to predict future learning performance. This provides evidence that understanding how learners use SMART processes during a task is essential to identify learning performance. Such evidence provides a first step in informing more intelligent, adaptive scaffolds for SRL. It is important to note however, that within Hutt et al. (2021) study, multimodal data could not capture when learners used *rehearsal* operations. This limitation may be due to the difficulty in capturing SRL operations in real time during learning, especially as such operations can encompass several different behaviors within a single learning session or are difficult for learners to deploy due to the complexity of the information, the task, and the SRL operation.

These prior studies on Winne (2018) SMART operations show that traditional methods such as self-report measures can capture learners’ perceptions of their SRL abilities and deployment of strategies but are limited to learners’ knowledge, understanding, and accurate reflection of their SRL. Additionally, the use of a single data channel to capture these SRL SMART operations limits researchers’ ability to accurately detect and identify behaviors to triangulate instances of operation use. As such, to capture learners’ deployment of SRL operations during game-based learning, multimodal data should be captured and analyzed (Azevedo et al., 2019). We argue that researchers’ examination of learners’ SMART operations during learning should incorporate a temporal element, expanding the methodologies of previous studies (Hutt et al., 2021; Zhang et al., 2022). The current study examined how learners’ sequential transitions across SMART operations during learning changed depending on the amount of agency afforded to learners during the task and how this informed learners’ understanding of the content.

## 4. Current study

The current study aims to identify how restricted agency during game-based learning scaffolds learners’ deployment of SRL SMART operations and how these operations are related to learning outcomes. To achieve this goal, this study examines how learners differing in the degree of scaffolding, embedded within Crystal Island, temporally deploy SMART operations during game-based learning and how each of the sequential transitions between individual SMART operations relate to learning outcomes. This study asked three research questions: (1) Are there differences in the frequency proportions of SMART operation deployment during game-based learning between agency conditions?; (2) Are there differences in the way learners transitioned between SMART operations during game-based learning across agency conditions?; and (3) To what extent do the probabilities of learners’ SRL SMART operation transitions relate to learning gains and agency conditions?

For the first research question, we hypothesize that learners who are restricted in their agency will demonstrate a greater number of SMART operations than learners who do not receive scaffolding during game-based learning. For the second research question, we hypothesize that learners who are scaffolded during game-based learning will demonstrate greater transition probabilities across all SMART operations as they dynamically deploy SRL more often and effectively than learners who are not scaffolded. Lastly, for the third research question, we hypothesize that learners who are scaffolded will demonstrate a stronger relationship between SRL SMART operations and learning outcomes where the probability that they transition across SMART operations will be more positively related to learning gains. All hypotheses follow prior literature in which restricted agency as a scaffold has been shown to increase learning outcomes (Taub et al., 2019, 2020; Cloude et al., 2021) and support SRL processes (Dever et al., 2021, 2022), and SMART operation use has been associated with increased learning outcomes (Hutt et al., 2021; Zhang et al., 2022).

## 5. Methods

### 5.1. Participants

A total of 120 undergraduate students from a North American public university were recruited to play Crystal Island. However, due to data loss, 26 students were removed from the analyses which resulted in a total of 94 participants (Age Range: 18–26, *M* = 20.0, *SD* = 1.65; 67% female) whose data were used to answer our research questions. The 94 participants were randomly assigned to one of two scaffolding conditions: the Full Agency condition (*N* = 56) and the Partial Agency condition (*N* = 38) in which participants were afforded varying levels of agency (see Embedded Scaffolding Conditions section for more details). Demographic questionnaires administered prior to the Crystal Island task revealed that the majority of participants reported that they do not or rarely play video games (55%), have average, limited, or no video game playing skills (73%), and play between 0 to 2 h of video games every week (68%). Participants were compensated $10 per hour, up to $30, for participating in this study. This study was approved by North Carolina State University’s Institutional Review Board for the Use of Human Subjects in Research (Protocol#: 5623).

### 5.2. Crystal Island environment

**Figure 1:**
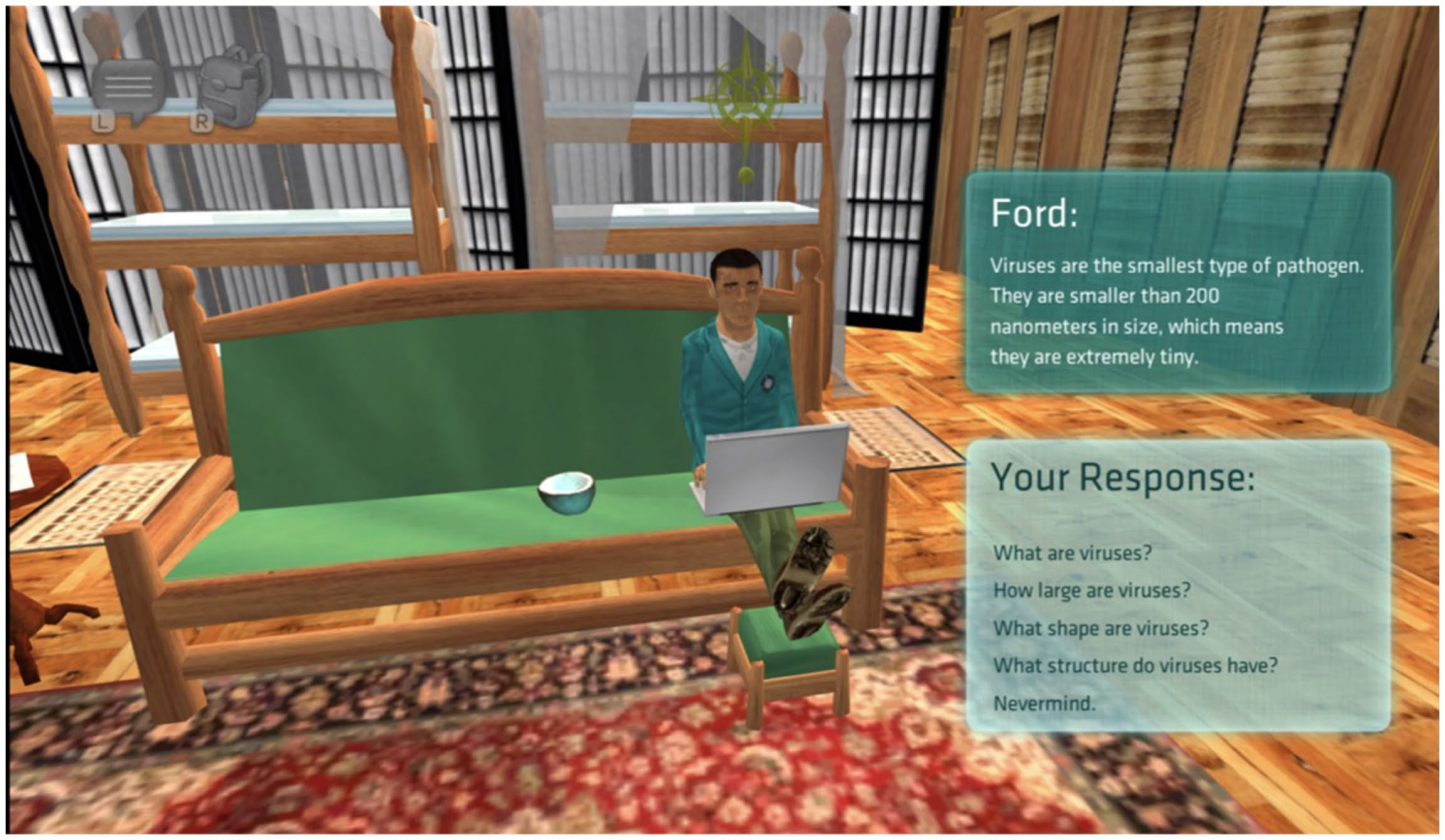
Crystal Island NPC dialog with participants. Screenshots from Crystal Island adapted with permission from JL from North Carolina State University IntelliMEDIA Group (https://www.intellimedia.ncsu.edu/about/).

Crystal Island: Lost Investigation is an immersive narrative-centered GBLE designed to foster the use of scientific reasoning while problem solving, improve engagement with science topics, and help students gain content knowledge about microbiology (Rowe et al., 2011; Dever et al., 2022). Within this GBLE, learners assume the role of an epidemic expert undertaking the responsibility of diagnosing an unknown ailment afflicting their fellow researchers stationed on a remote volcanic island. The central gameplay revolves around solving a mysterious ailment, collecting data by talking to other sick research team members and analyzing clues, researching details about how viruses, bacteria, and other illness-causing infections develop, spread, and can be cured, and self-testing their new game and content knowledge. To successfully complete the game, learners are required to diagnose the mystery ailment (e.g., influenza, smallpox, salmonellosis), provide a suitable treatment (e.g., vaccination, relaxation, other preventive measures), and determine the origin of that outbreak (e.g., from the contaminated food and drink such as bread, apple, milk, etc.). This requires that learners engage in dialog with non-player characters (NCPs) who supply pertinent information related to a subject matter (e.g., what are bacteria, their shape, size, and characteristics; see Figure 1) or steps that can aid in solving the puzzle (e.g., symptoms).

**Figure 2:**
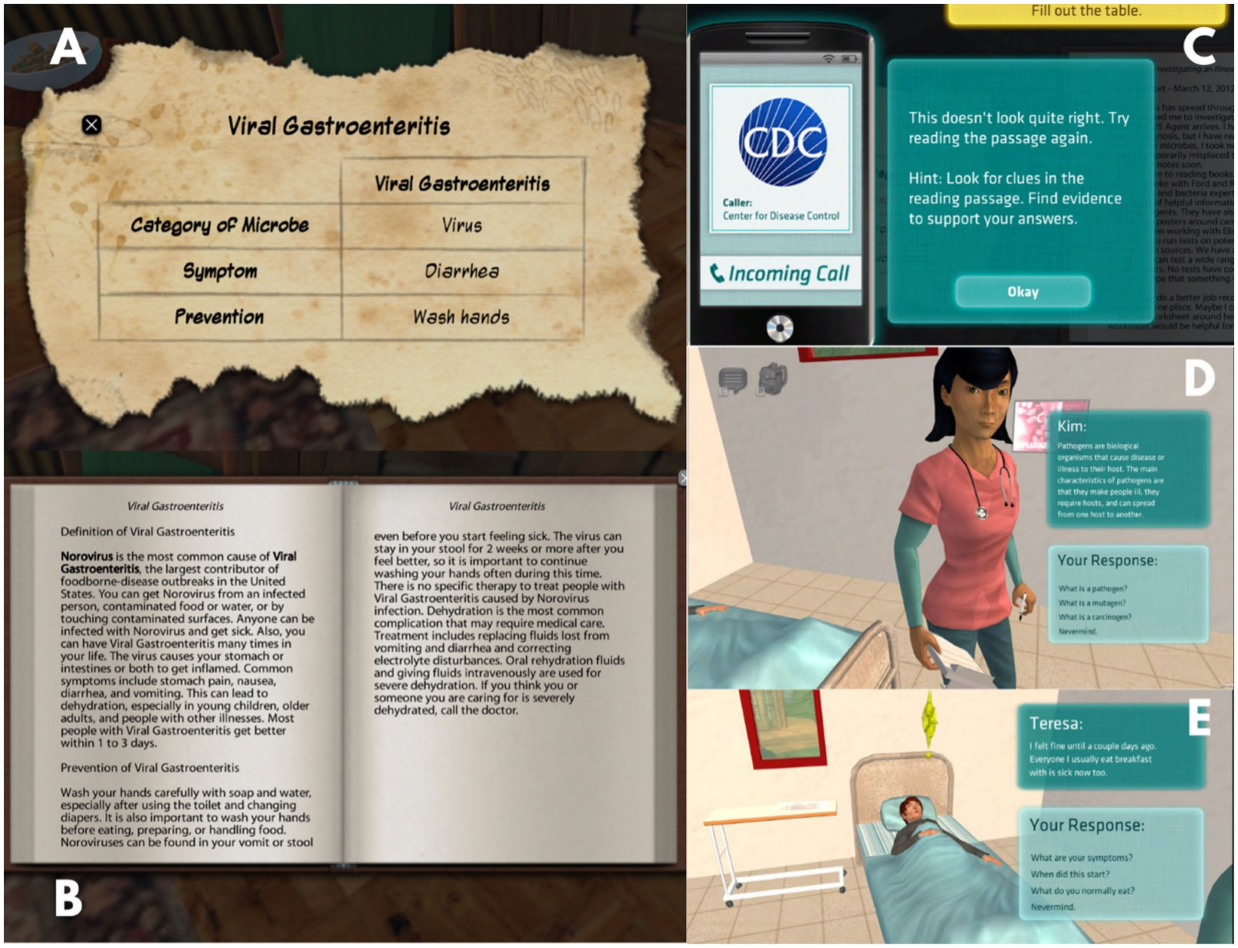
Screenshots of elements in Crystal Island; **(A)** concept matrix; **(B)** book; **(C)** feedback on concept matrix; **(D)** example of dialog with the camp nurse, an NPC; **(E)** patient conveying symptoms. Screenshots from Crystal Island adapted with permission from JL from North Carolina State University IntelliMEDIA Group (https://www.intellimedia.ncsu.edu/about/).

Additionally, learners have access to informational content presented in the form of books and research articles spread throughout the remote island. These resources provide critical microbiology information that may be crucial for the successful completion of the investigation. Concept matrices act as evaluative metrics (Figure 2A) of participants’ understanding and application of the information extracted from the corresponding text (Figure 2B). If the learner does not get the correct answer, the system provides feedback and asks the learner to reread the passage (Figure 2C). Learners are not required to complete or interact with the concept matrix, unless they are in the Partial Agency condition. If a learner fails to correctly answer the concept matrix within three attempts, the game prompts the learner to move on. In addition to these informational texts, learners must also collect and document data on the symptoms of the mystery illness. To do so, they must talk to the camp nurse (Figure 2D) as well as sick residents about their symptoms (Figure 2E), what residents were doing prior to becoming sick, and then explore the island to collect potential contaminates for testing.

**Figure 3:**
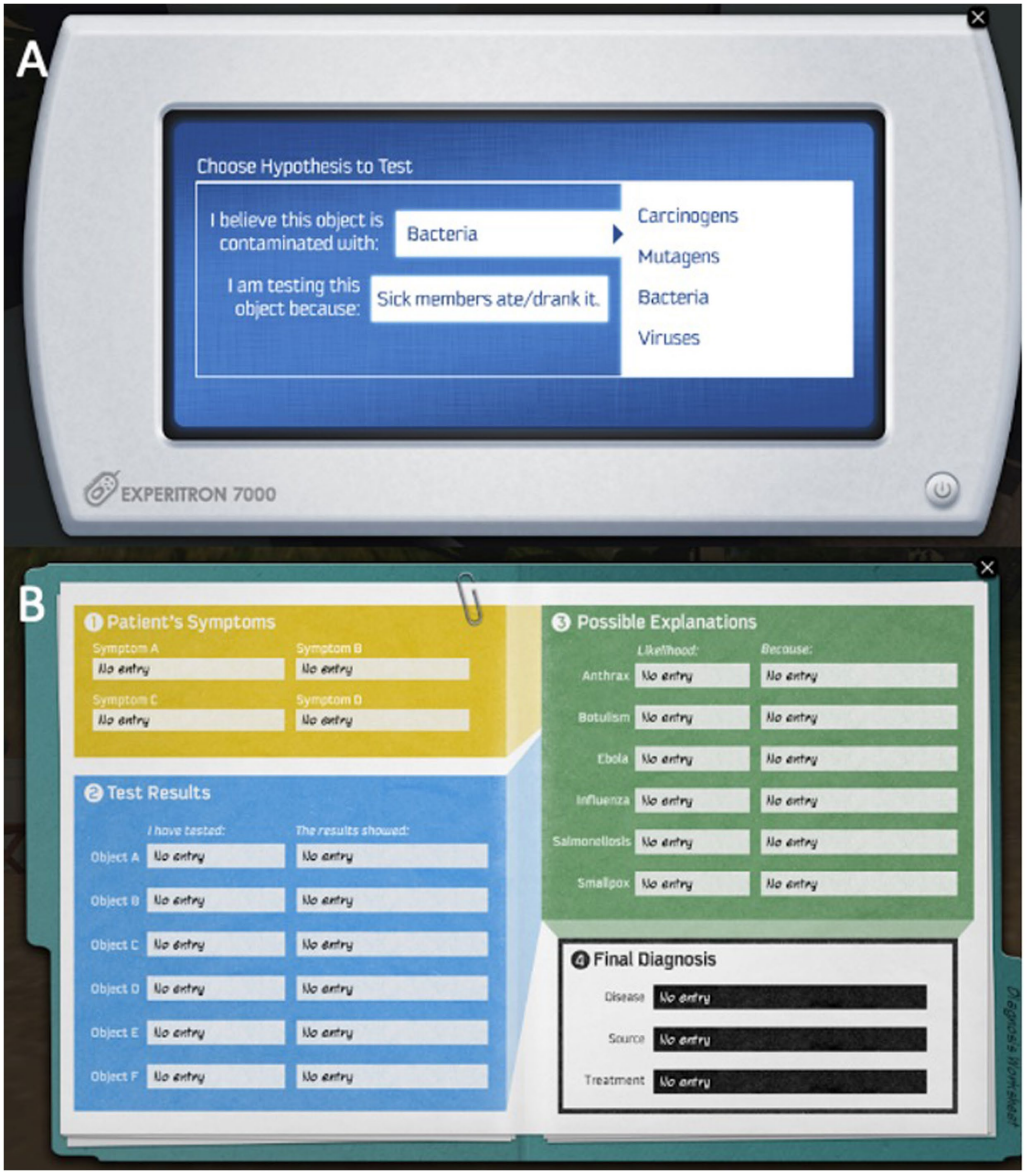
Crystal Island screenshot of elements; **(A)** scanner and hypothesis generation; **(B)** diagnostic worksheet. Screenshots from Crystal Island adapted with permission from JL from North Carolina State University IntelliMEDIA Group (https://www.intellimedia.ncsu.edu/about/).

A lab scanner (Figure 3A) is made available to learners for testing hypothesized contagions across different food items that can be gathered throughout the environment. These hypotheses are then translated into the final diagnosis within the diagnostic worksheet (see Figure 3B). To successfully complete the game, participants must successfully identify and submit the disease, the transmission of the disease, and the appropriate treatment to Kim, the NPC camp nurse via the diagnostic worksheet.

#### 5.2.1. Embedded scaffolding conditions

Learners were randomly assigned to one of three agency conditions which impacted how they could interact with Crystal Island. The ‘Full Agency’ condition provided participants total agency by allowing them the freedom to initiate any actions without restrictions during their learning experience. The “Partial Agency” condition-imposed limitations on participants’ actions by setting an optimal path that they needed to adhere to for the successful completion of the mystery. For instance, participants had to explore the camp and visit specific buildings (such as the infirmary, camp kitchen, lab center, etc.) in a predetermined sequence designed to optimize information acquisition. Finally, participants in the “No Agency” condition had a vicarious learning experience by observing a playthrough of the game from a third-person perspective, devoid of any interaction with game elements or the capacity to manipulate the playthrough video (e.g., play, pause). As participants were not able to interact with the game themselves, we have excluded these participants from our current study.

### 5.3. Experimental procedure

**Figure 4:**
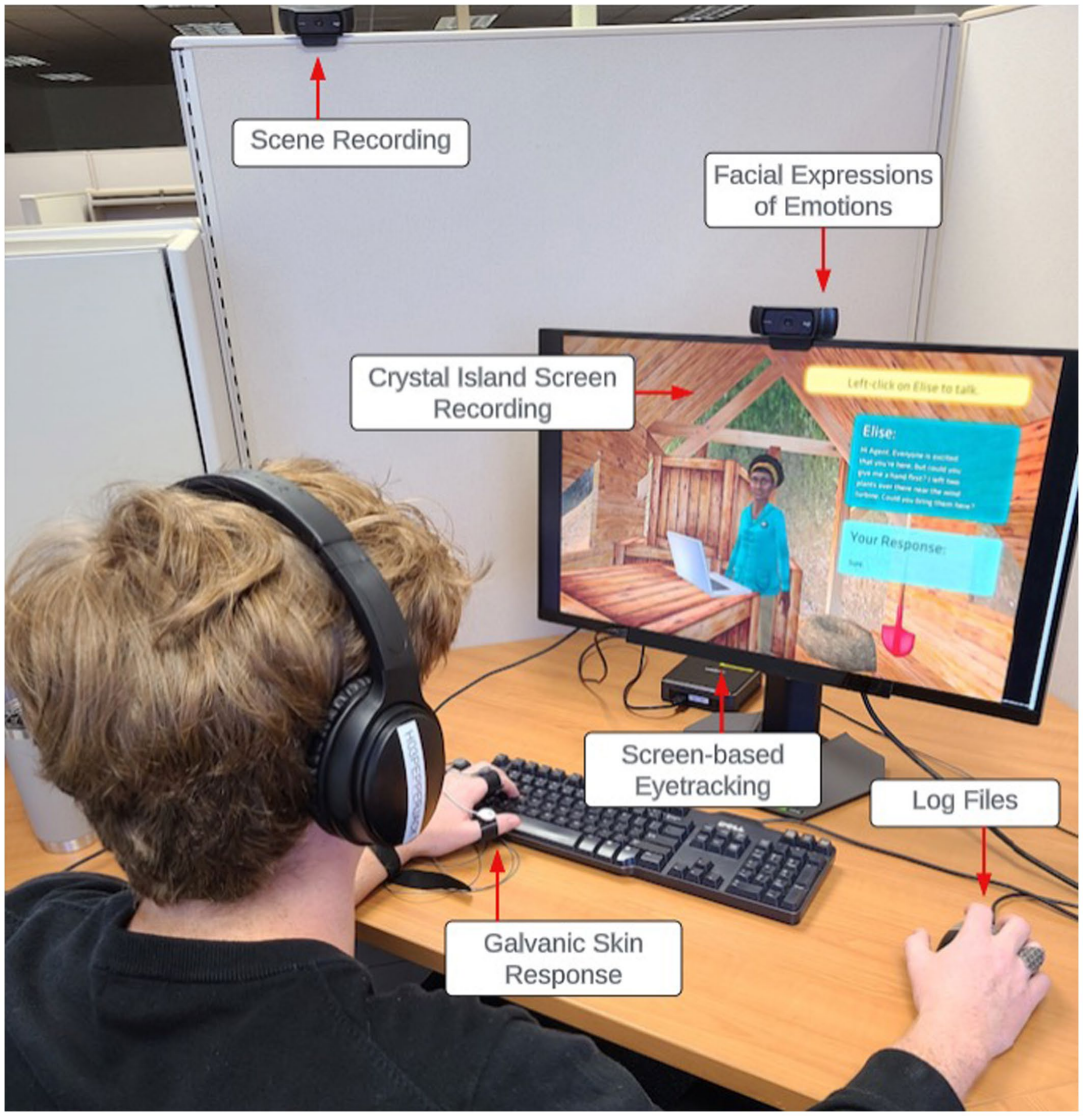
Experimental setup.

Following informed consent, participants completed a battery of self-reports and questionnaires about demographics, microbiology content knowledge, emotions (Achievement Emotions Questionnaire; Pekrun et al., 2011) and motivation (Achievement Goals Questionnaire; Elliot and Murayama, 2008). The microbiology content knowledge pre-test included 21, 4-option multiple choice questions The questions incorporated within the pretest questionnaire cover a broad spectrum of topics ranging from what microbiology is, its purpose to cellular morphology to detecting a genetic aliment from a provided list of symptoms. Following the completion of the pretest, participants underwent calibration with the SMI EYERED 250 eye tracker, using a precise 9-point calibration process. Eye-tracking data collected participants’ fixations, which are relatively stable gaze behaviors on a single area of interest. These fixations were then used to calculate participants/ dwell times on areas of interest to identify when, for how long, and the frequency of participants’ attention toward an object within the Crystal Island environment. Subsequently, for calibration accuracy, participants were instructed to maintain a neutral facial expression and composure during the calibration process with both the facial recognition of emotions software and the electrodermal bracelet to measure galvanic skin response. This calibration established a baseline captured using the Attention Tool 6.3 (shown in Figure 4 of participant setup). For the purposes of this study, we did not examine facial expressions or physiological data.

Following the calibration process, participants begin their learning experience with Crystal Island beginning with a tutorial to teach participants how to move around the island and interact with various elements. During the tutorial phase of the game, learners (participants) were reminded of the essentiality of employing a variety of resources such as books, virtual posters, research articles, and interacting with non-player characters while engaging in game-based learning. Throughout their engagement with Crystal Island, we documented their process data, which spanned various factors like eye movements (e.g., fixation and saccade), emotional facial expressions (e.g., joy, neutral, frustration), and log files (e.g., duration of time spent in participating in the activity). However, for this study, we have only examined participant’s eye movements and log files. Upon accurately solving the mystery, participants were given a posttest designed to evaluate discrepancies in their microbiology knowledge and several self-report questionnaires. These questionnaires included the same pre-test questionnaires in addition to the Intrinsic Motivation Inventory (Ryan et al., 1983), the Perceived Interest Questionnaire (Schraw et al., 1995), and the Presence Questionnaire (Witmer and Singer, 1998). Upon completion of the study, the researcher conducted a debriefing session, provided monetary compensation, and thanked participants for their involvement and time. It is important to note that the aforementioned self-reports and facial expressions of emotions were included for replicability purposes and were not used for addressing the research questions within this study. The data used to support the research questions included eye movements, log files, and performance data from microbiology knowledge pre- and post-tests.

### 5.4. Apparatus

As participants completed the Crystal Island task, several trace data were collected including eye tracking, facial expressions of emotions, galvanic skin response, and log files. For the purposes of these research questions, only eye tracking and log files were analyzed. An SMI RED250 eye tracker was used to collect and contextualize participants’ eye gaze behaviors. Specifically, eye-tracking data identified where participants were looking at the screen, contextualized the location of participants’ gaze to the Crystal Island environment, and recorded at what time these gazes occurred. Actions captured using eye-tracking data included when participants were reading books and research articles and when participants edited and completed concept matrices. Log files were used to identify when a participant started the game, the actions they took while completing the game, and the time at which actions were taken. Actions captured by log files included movement across pre-defined areas, viewing posters, filling out and submitting the worksheet, conversing with NPCs, and scanning and hypothesizing about food items. Eye-tracking and log-file data were aligned using iMotions Attention Tool 6.2 software (iMotions, 2016) which ordered the actions according to the timestamps.

### 5.5. Coding and scoring

#### 5.5.1. SRL operations

Actions that participants could take while playing Crystal Island were classified into a *SMART Operation*, captured using log-file and eye-tracking data (see Table 1). We argue that as participants choose to engage in these activities within Crystal Island, these activities elicit SMART operations that assist participants in using SRL processes to achieve their goals. *Searching* was identified by participants’ movements across location boundaries. For example, if participants left the clinic and entered another building without taking any other action, this was counted as two sequential movements. By completing and submitting concept matrices, participants demonstrate a *Monitoring* operation, specifically a judgment of learning. In reading books and research articles, viewing posters, filling out worksheets, conversing with NPCs, and hypothesizing about diseases, participants are engaging in *Assembling/rehearsing* operations as they gather information, rehearse that information in working memory, and coordinate multiple sources of information to create a full mental model of microbiology from instructional materials. When participants submitted their final diagnosis, these actions were labeled as *Translating* operations as the participant took learned information from instructional materials and contextually applied that information.

**Table 1:** Crystal Island actions captured and classified into SMART operations.

SMART operation	Action	Data capture methodology
Searching	Movement across pre-defined boundaries	Log Files
Monitoring	Submission of concept matrices	Eye Tracking
Assembling/rehearsing	Viewing posters, books, and research articles	Eye Tracking & Log Files
	Talking with NPCs	Log Files
	Filling out the Diagnostic Worksheet	Log Files
	Scanning food items for diseases	Log Files
Translating	Submitting a final diagnosis	Log Files

#### 5.5.2. Transition probabilities

**Figure 5:**
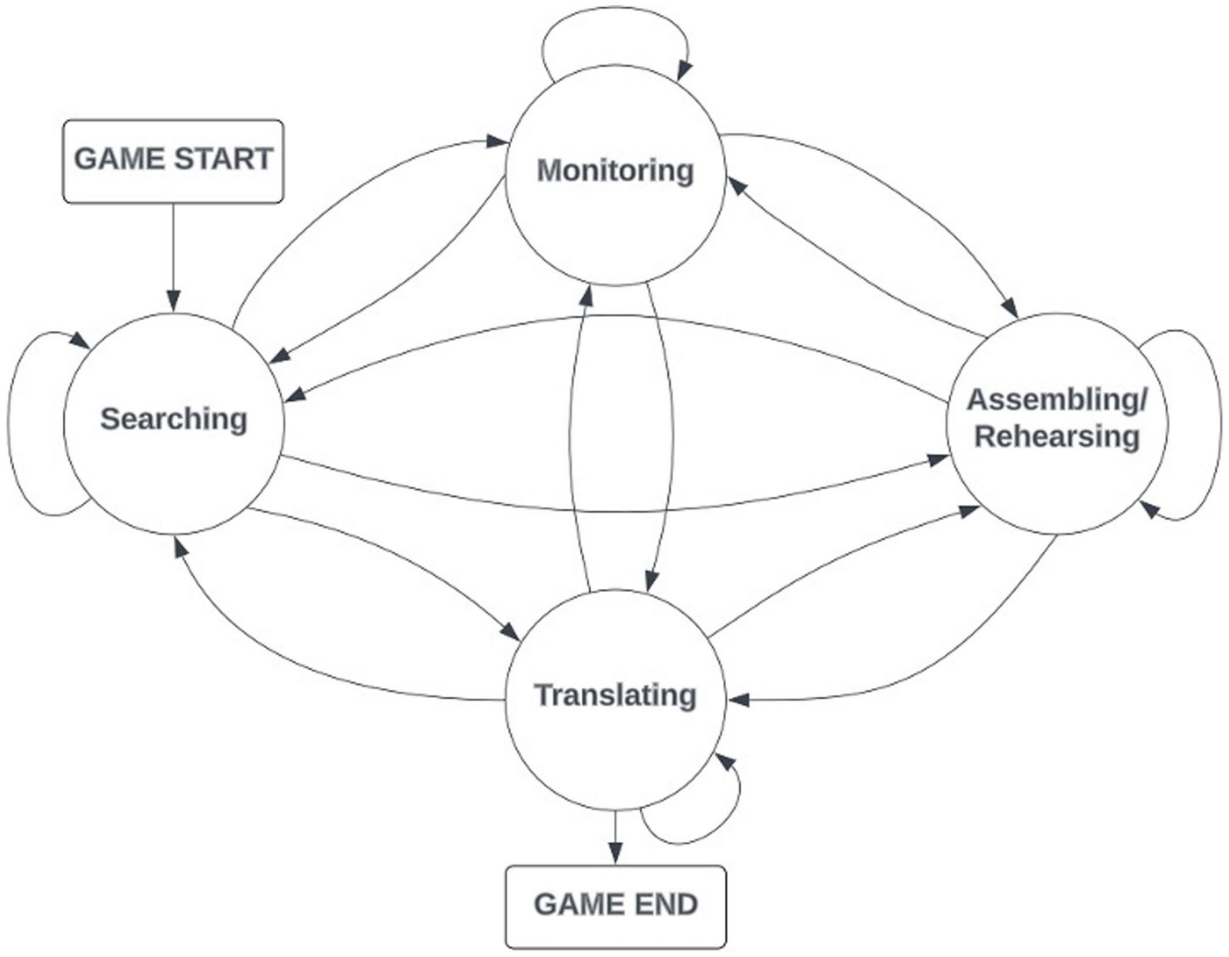
Possible data-driven transitions across SMART operations.

A transition matrix for each participant was calculated based on the sequential operations derived from the aligned eye-tracking and log file data via timestamps. These transition matrices identified the probability of a transition from one SMART operation to another (e.g., Searching to Monitoring). A total of 16 data-driven transitions were possible (see Figure 5).

#### 5.5.3. Learning gains

*Learning gains* were calculated using Marx and Cumming (2007) normalized change score equations in which participants’ differences in their pre- and post-task microbiology quizzes were identified while controlling for prior knowledge.

### 5.6. Data analysis

Several analyses were conducted to fully understand how agency as a scaffold relates to how learners deploy SRL SMART operations during game-based learning. Table 2 refers to how each research question was addressed by the analyses included within this paper and the overarching objective of including these analyses in supporting the research question.

**Table 2:** Analyses used to address each research question and their objectives.

Research question	Analysis	Objective
**RQ1.** Are there differences in the frequency proportions of SMART operation deployment during game-based learning between agency conditions?	2-way ANOVA (with pairwise *t*-tests) Between-Subjects: Agency Condition Within-Subjects: SMART Operation Dependent Variable: Frequency of SMART Operations	Examine the differences in frequencies of different SMART operations between agency conditions
**RQ2.** Are there differences in the way learners transitioned between SMART operations during game-based learning across agency conditions?	Four 2-way ANOVAs (with pairwise *t*-test) Between-Subjects: Agency Condition Within-Subjects: SMART Operation Dependent Variable: Transition Values of SMART Operation	Identify differences between agency conditions in learners’ transitions across SEARCHING operations
		Identify differences between agency conditions in learners’ transitions across MONITORING operations
		Identify differences between agency conditions in learners’ transitions across ASSEMBLING/REHEARSING operations
		Identify differences between agency conditions in learners’ transitions across TRANSLATING operations
**RQ3.** To what extent do the probabilities of learners’ SRL SMART operation transitions relate to learning gains and agency conditions?	Four Multiple Linear Regressions Predictor Variables: (1) Transitions into SMART Operations; (2) Agency Condition Outcome Variable: Learning Gains	Examine how transitions into SEARCHING operations relate to agency conditions and learning gains
		Examine how transitions into MONITORING operations relate to agency conditions and learning gains
		Examine how transitions into ASSEMBLING/REHEARSING operations relate to agency conditions and learning gains
		Examine how transitions into TRANSLATING operations relate to agency conditions and learning gains

### 5.7. Preliminary analyses

We first examine differences in participants’ learning gains between agency conditions to holistically understand how scaffolding relates to learning outcomes. An independent *t*-test found significant differences in learning gains between agency conditions [*t*(79.2) = −2.24, *p* < 0.05] in which participants in the Partial Agency condition (*M* = 0.33, *SD* = 0.24) demonstrated significantly greater learning gains than participants in the Full Agency condition (*M* = 0.22, *SD* = 0.24). This shows that restricted agency is a successful scaffold of learning outcomes in terms of learning domain content related to microbiology. This difference establishes a need to understand how restricted agency impacts SRL SMART operation deployment during game-based learning.

On average, the completion time for participants in the Full Agency condition is within 82.6 min (*SD* = 22.8). In contrast, participants assigned to the Partial Agency condition took an average of 96.8 min (*SD =* 18.7) minutes to complete the game, a significantly longer amount of time [*t*(88.7) = −3.33, *p < 0.05*]. While completion times varied across different conditions, no temporal constraints were imposed on participants within their respective environments. To account for this difference in task completion time, we examined the relative proportion of time participants spent engaging with each SMART operation to answer Research Question 1. In other words, we divided the raw frequency in which participants used Searching, Monitoring, Assembling/Rehearsing, and Translating operations by the total time each participant spent on task to identify the frequency proportions of SMART operations relative to how much time they spent on task. For Research Questions 2 and 3, in using transition probabilities, we account for this difference in time in which each probability is a proportion in which the transition probabilities are in relation to frequency of actions and transitions between actions across participants.

## 6. Results

### 6.1. Research question 1: are there differences in the frequency proportions of SMART operation deployment during game-based learning between agency conditions?

A two-way ANOVA (skew and kurtosis < |2|) was conducted to examine the differences in the frequencies of SMART operation deployment between agency conditions. Frequency proportions across all participants (*N* = 374) ranged from 0 to 0.07 (*M* = 0.02, *SD* = 0.01). Results revealed significant main effects of condition [*F*(1, 368) = 49.2, *p* < 0.01] and SMART operations [*F*(3, 368) = 244.4, *p* < 0.01]. Across all SMART operations, participants within the Full Agency condition engaged in a significantly greater frequency proportion of SMART operations (*M* = 0.02, *SD* = 0.01) than those in the Partial Agency condition (*M* = 0.01, *SD* = 0.01). Pairwise *t*-tests with Bonferroni corrections (*p* < 0.0083) for six tests found significant differences across SMART operations in which participants engaged in significantly more Assembling/Rehearsing operations (*M* = 0.03, *SD* = 0.01) followed by Searching (*M* = 0.02, *SD* = 0.01) and Monitoring (*M* = 0.02, *SD* = 0.01) and significantly less Translating operations (*M* = 0.0, *SD* = 0.0; see Table 3 for statistics). The two-way ANOVA also revealed a significant interaction effect between frequency proportions of SMART operations and conditions [*F*(3, 368) = 22.3, *p* < 0.01]. However, post-hoc analyses with Bonferroni corrections (*p* < 0.0.125) found that the only significant difference between condition across the frequency proportions of SMART operations were related to participants’ Searching operations in which participants in the Full Agency condition deployed significantly more searching operations (*M* = 0.03, *SD* = 0.01) than participants in the Partial agency condition [*M* = 0.01, *SD* = 0.0; *t*(72.2) = 10.6, *p* < 0.01].

**Table 3:** Differences in participants’ SMART operations use during learning.

SMART operation	*M*	*SD*	1	2	3
Searching	0.02	0.01			
Monitoring	0.02	0.01	1.37		
Assembling/rehearsing	0.03	0.01	−4.57*	−7.11*	
Translating	0.0	0.0	17.4*	21.9*	30.4*

* indicates significant t-value at *p* < 0.0083.

### 6.2. Research question 2: are there differences in the way learners transitioned between SMART operations during game-based learning across agency conditions?

**Figure 6:**
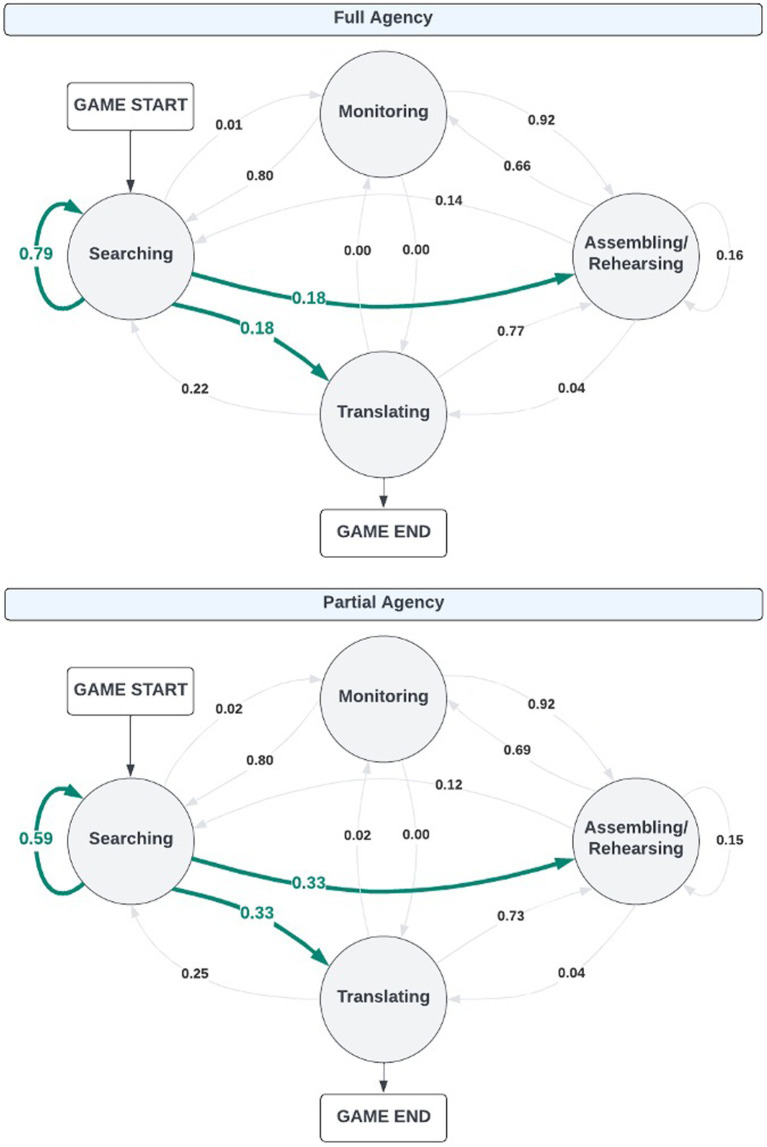
Differences between conditions across SMART operation transitions. Bold green transition line indicates significant difference between agency conditions.

For this research question, we calculated a transition matrix for each participant to examine how participants sequentially deployed SMART operations during game-based learning. To do so, we used all participants’ log-file and eye-tracking data to identify which SMART operation was deployed at what time. Participants received probability scores for 16 possible transition states (e.g., searching to searching, searching to monitoring). Figure 6 represents the average probability that the transition occurred between each agency condition. Transitions marked in green highlight transitions that had significant differences between conditions.

Four two-way ANOVAs were conducted to identify the differences between conditions in learners’ transitions to: (1) Searching (e.g., monitoring *to* searching); (2) Monitoring (e.g., translating *to* monitoring); (3) Assembling/Rehearsing (e.g., searching *to* assembling/rehearsing); and (4) Translating (e.g., searching *to* translating). Given repeated tests (*N* = 4), significance was corrected using Bonferroni such that significant effects were *p* < 0.0125.

*Searching.* The first two-way ANOVA (see Table 4) examined differences in the deployment of SMART operations to Searching operations between agency conditions. There were main effects of both SMART operations [*F*(3,368) = 724.0, *p* < 0.0125] and condition [*F*(1,368) = 17.5, *p* < 0.0125] as well as a significant interaction effect in which the transition probabilities significantly differed between agency conditions and across SMART operations [*F*(3,368) = 21.6, *p* < 0.0125]. Comparisons between conditions found that participants had significantly greater transitions to Searching operations when they were in the Full Agency condition (*M* = 0.31, *SD* = 0.3) than participants in the Partial Agency condition (*M* = 0.26, *SD* = 0.23) with a statistically significant difference in participants’ recursive Searching transitions (i.e., Searching to Searching; *t* = −5.86, *p* < 0.0125) between the Full (*M* = 0.79, *SD* = 0.05) and Partial (*M* = 0.59, *SD* = 0.06) Agency conditions.

**Table 4:** Searching two-way ANOVA summary table.

Variable	*df*	SS	MS	*F* value	Value of *p*
to Monitoring transition	3	23.8	7.92	724.0	*p* < 0.0125*
Agency condition	1	0.19	0.19	17.5	*p* < 0.0125*
Interaction	3	0.71	0.24	21.6	*p* < 0.0125*
Residuals	368	4.02	0.01		

* indicates significant F-value at *p* < 0.0125.

***Monitoring.*** The next two-way ANOVA (see Table 5) examined differences in the deployment of SMART operations to Monitoring operations between agency conditions. While the model revealed significant main effects of SMART operations [*F*(2,276) = 1952.2, *p* < 0.0125] and condition [*F*(1,276) = 4.68, *p* < 0.05] in which participants in the Full Agency condition (*M* = 0.18, *SD* = 0.05) had lower probabilities of transitioning into Monitoring operations from other SMART operations than those in the Partial Agency condition (*M* = 0.33, *SD* = 0.07), there was no significant interaction effect (*p* > 0.05).

**Table 5:** Monitoring two-way ANOVA summary table.

Variable	*df*	SS	MS	*F* value	Value of *p*
to Monitoring transition	2	27.1	13.6	1952.17	*p* < 0.0125*
Agency condition	1	0.03	0.03	4.679	*p* < 0.0125*
Interaction	2	0.003	0.001	0.197	*p* > 0.0125
Residuals	276	1.92	0.01		

* indicates significant F-value at *p* < 0.0125.

*Assembling/Rehearsing*. The third two-way ANOVA (see Table 6) aimed to identify differences in the deployment of SMART operations to Assembling/Rehearsal operations between agency conditions. Results from this ANOVA showed a significant main effect of SMART operations [*F*(3,368) = 1099.6, *p* < 0.0125] and a significant interaction effect [*F*(3,368) = 14.0, *p* < 0.0125] where, although there was no main effect of condition, participants in the Full and Partial Agency conditions differed in their transitions to Assembling/Rehearsing operations across SMART operations. Specifically, comparisons between conditions across these transition probabilities found that participants in the Full Agency condition demonstrated significantly lower probabilities of transitions from Searching to Assembling/Rehearsing (*M* = 0.18, *SD* = 0.05) than participants in the Partial Agency condition (*M* = 0.33, *SD* = 0.07; *t* = 5.00, *p* < 0.0125).

**Table 6:** Assembling/rehearsing two-way ANOVA summary table.

Variable	*df*	SS	MS	*F* value	Value of *p*
to Monitoring transition	3	40.2	13.4	1099.6	*p* < 0.0125*
Agency condition	1	0.05	0.05	3.80	*p* > 0.0125
Interaction	3	0.51	0.17	14.0	*p* < 0.0125*
Residuals	368	4.48	0.01		

* indicates significant F-value at *p* < 0.0125.

*Translating.* The last two-way ANOVA (see Table 7) examined differences in the deployment of SMART operations to Translating operations between agency conditions. Results found a significant main effect of SMART operation [*F*(2,276) = 1049.4, *p* < 0.01] with a significant interaction effect [*F*(2,276) = 115.3, *p* < 0.0125] in which participants in the Full Agency condition demonstrate significantly lower probabilities in their transitions to Translating operations from Searching operations (*M* = 0.18, *SD* = 0.05) than participants in the Partial Agency condition (*M* = 0.33, *SD* = 0.07; *t* = 13.3, *p* < 0.0125).

**Table 7:** Translating two-way ANOVA summary table.

Variable	*df*	SS	MS	*F* value	Value of *p*
to Monitoring transition	2	3.09	1.54	1049.4	*p* < 0.0125*
Agency condition	1	0.17	0.17	115.3	*p* < 0.0125*
Interaction	2	0.34	0.17	115.6	*p* < 0.0125*
Residuals	276	0.41	0.002		

* indicates significant F-value at *p* < 0.0125.

In sum, across all transition probabilities, participants in the Full Agency condition demonstrated greater recursive Searching operations than those in the Partial Agency condition. This indicates an inefficiency of action use in which participants without scaffolding were searching for information rather than reading information, engaging in monitoring strategies, etc. This also indicates that participants without scaffolding needed more instruction on how to navigate the environment to engage in efficient use of (game-based) environment features. Further, participants in the Partial Agency condition demonstrated significantly greater transitions from Searching to both Assembling/Rehearsing and Translating operations. This demonstrates a greater adaptivity of SRL SMART operations when participants were scaffolded via restricted agency.

Research Question 3: To what extent do the probabilities of learners’ SRL SMART operation transitions relate to learning gains and agency conditions?

*Searching.* Correlations were identified between learning gains and SMART operation transitions to Searching operations to identify how transition probabilities relate to learning gains. Searching to Searching [*r*(92) = −0.25, *p* < 0.05] as well as Assembling/Rehearsing to Searching [*r*(92) = −0.20, *p* < 0.05] transition probabilities were found to be significantly and negatively related to learning gains. A multiple linear regression was then conducted to identify how these transition probabilities and agency conditions interact with each other to predict learning gains. The regression was not significant (*p* > 0.05), indicating that transitions from SMART operations to Searching operations are not significant predictors of learning gains.

*Monitoring.* Correlations between learning gains and SMART operation transition probabilities to Monitoring operations. Results found a significant, positive relationship between the transition probability from Assembling/Rehearsing to Monitoring operations and learning gains [*r*(92) = 0.21, *p* < 0.05]. A multiple linear regression examined how agency conditions and Assembling/Rehearsing to Monitoring operation transition probabilities related to learning gains. Overall, the linear regression was significant [*F*(2,91) = 4.31, *p* < 0.05; *R*^2^ = 0.12]. While results from this model did not find a significant main effect of the transition probability (*p* > 0.05), results did find a significant interaction effect between this transition probability and learning gains (*t* = 2.03, *p* < 0.05) where as participants in the Partial Agency condition demonstrated greater transition probabilities from Assembling/Rehearsing operations to Monitoring operations, learning gains increased at a greater rate than when participants were in the Full Agency condition. In sum, scaffolding via agency promotes participants’ transitions from Assembling/Rehearsing to Monitoring which further increases learning gains.

*Assembling/Rehearsing.* Correlations were conducted to examine the relationship between learning gains and SMART operation transition probabilities to Assembling/Rehearsing operations. While correlations found a significant positive relationship between learning gains and the transition probability from Searching to Assembling/Rehearsing operations [*r*(92) = 0.25, *p* < 0.05], a multiple linear regression using this transition probability and agency as predictor variables did not reveal significant effects on learning gains (*p* > 0.05).

*Translating.* Correlations between learning gains and the probability that participants transitioned from a SMART operation to a Translating operation found significant relationships between learning gains and when learners transition from Assembling/Rehearsing [*r*(92) = −0.21, *p* < 0.05] and Searching [*r*(92) = 0.25, *p* < 0.05] operations to Translating operations. These two transition probabilities were used as predictor variables along with agency conditions to examine their effect on participants’ learning gains within a multiple linear regression. Overall, the model was significant [*F*(4,89) = 2.88, *p* < 0.05; *R*^2^ = 0.11] where results showed a significant interaction effect of Assembling/Rehearsing to Translating probabilities and condition (*t* = −2.15, *p* < 0.05). As participants in the Full Agency condition demonstrated greater transition probabilities from Assembling/Rehearsing to Translating operations, learning gains decreased at a greater rate compared to learners in the Partial Agency condition.

In sum, results from this research question showed that learning gains increased when participants in the Partial Agency condition demonstrated greater transition probabilities from Assembling/Rehearsing operations to Monitoring operations. Additionally, when participants in the Full Agency condition demonstrated greater transition probabilities from Assembling/Rehearsing to Translating operations, learning gains decreased. This may be due to either the pre-mature application of information that has been found during Assembling/Rehearsing to other contexts or due to the lack of other processes that facilitate a successful transition between these operations (e.g., Monitoring operation).

## 7. Discussion

The goal of this study was to use multimodal data to understand how learners’ sequential transitions across SMART operations were related to the level of scaffolding received during game-based learning and how this contributed to learners’ overall learning gains. The *first research question* examined the differences between conditions in the proportion in which each SMART operation was deployed during learning. Results found that learners engaged in Assembling/Rehearsing operations more often, followed by Searching and Monitoring operations, and lastly followed by Translating operations and that generally, learners who received scaffolding engaged in significantly less SMART operations than learners in the full agency condition. While this may seem to indicate that agency as a scaffold discourages learners’ deployment of SRL SMART operations and is not consistent with hypotheses and prior literature (Dever et al., 2021, 2022; Hutt et al., 2021; Zhang et al., 2022), results further found that only the Searching operation significantly differed between conditions where learners in the Full Agency condition had a significantly greater proportion of Searching operations than those in the Partial Agency condition. As Searching was identified as the movement across pre-defined boundaries within the game environment, we interpret this finding to mean that scaffolding learners by limiting their agency supports learners’ exploration and navigation of the GBLE, leading to more efficient interactions with GBLE elements. This interpretation of findings is an important first step to understanding that the deployment of SMART operations is not, in and of itself, an ideal use of SRL, rather the balance of using SMART operations in accordance with the amount of time spent in the environment and in relation to other operations is a key component of understanding efficient and accurate SRL.

The *second research question* utilized learners’ transition probabilities across SMART operations to examine differences between agency conditions in how SMART operations were sequentially deployed. Results were partially consistent with hypotheses in which learners across both agency conditions demonstrated differences in their transition probabilities across SMART operations but were mixed in which group demonstrated greater or lower transition probabilities across specific SMART operations. Across all results within this research question, non-scaffolded learners compared to scaffolded learners had: (1) more recursive Searching transitions, consistent with findings from the first research question; (2) lower probabilities of transitioning into Monitoring operations from other SMART operations; (3) lower probabilities of transitions from Searching to Assembling/Rehearsing; and (4) lower probabilities in transitioning to Translating from Searching. These findings show that learners who are supported via restricted agency use a greater variety of SMART operations and transition more often between SMART operations than learners who were not scaffolded, demonstrating greater SRL balance and efficiency. This extends the SMART theoretical framework (Winne, 2018) as well as prior literature on promoting SRL to increase learning outcomes (Hutt et al., 2021; Zhang et al., 2022; Dever et al., 2023) to include a temporal understanding of how scaffolding can support learners’ transitions across SRL SMART operations.

The *third research question* was examined to further understand how learners deployed SMART operations relate to the scaffolding present within the GBLE and learning outcomes, building on the second research question. Results from this research question showed that while transitions from SMART operations to Search and Assembling/Rehearsing did not significantly relate to learning gains, there were significant relationships in the transitions to Monitoring and Translating operations. Specifically, learners who were scaffolded demonstrated greater transitions between Assembling/Rehearsing to Monitoring which were related to greater learning outcomes. Conversely, non-scaffolded learners who demonstrated greater transitions from Assembling/Rehearsing to Translating had significantly lower learning gains than learners who were scaffolded. This supports our hypotheses in which restricted agency as a scaffold aids learners in engaging in Monitoring operations after Assembling information throughout the environment and Rehearsing information in working memory. In comparing groups who received scaffolding and those who did not receive scaffolding, analyses also revealed that scaffolding learners during game-based learning mitigates the negative impacts of certain SMART operation transitions (i.e., Assembling/Rehearsing to Translating) on learning outcomes that otherwise would have been present. As such, this study reveals that the sequential transitions between certain SMART operations should be either encouraged or discouraged based on their relationships to learning operations, furthering our understanding of how learners should be optimally engaging in SRL SMART operations to increase learning outcomes.

## 8. Limitations

There are a few limitations with this study that reflect the pervasive limitations within game-based learning and SRL literature. Methodologically, while this paper classified SMART operations according to the direct actions that the participants took, we did not separate Assembling and Rehearsing operations but rather considered them as one action. Theoretically, these operations should be considered separate but with the data that was collected, log files and eye tracking methodologies cannot separate these processes. Specifically, these data cannot identify when a participant assembles information vs. rehearses information within working memory. This is a limitation seen in prior work by Hutt et al. (2021) in which the rehearsing operation could not be identified through the data collected. As such, this paper combined these processes in which an assembling action can reflect learners’ rehearsal of this information. To mitigate the impact of this limitation on generalizability and theoretical applications, we suggest future studies should collect concurrent verbalizations to capture these processes separately (see Azevedo et al., 2019).

Further, the transition probabilities within this paper were used to identify the probability that a transition between two states occurred. While further analyses can be conducted to identify the probability a transition occurred given the status of a previous state (e.g., the probability that the transition from A to B occurred given that action C preceded A), there stands the limitation that this analysis does not take the history of leaners’ prior use of SRL processes or time of session (and other potentially relevant instructional conditions) into account. In other words, these transition probabilities apply the same weight to transitions regardless of when the transition was deployed and what the learner has previously done within the GBLE. As such, future studies should attempt to understand how the history (i.e., temporal deployment of SRL processes) and prior actions completed by a learner may influence the transition probabilities over time.

## 9. Applications of findings

While the goal of this study was to use multimodal data to understand how learners transition across SRL SMART operations depending on the scaffolding provided to learners, there are several applications of the findings from this study to other methodologies and domains. For example, identifying transitions and its relationship to outcomes can support current literature on brain-computer interfaces (BCIs) and the effectiveness of such systems compared to their cost (Vourvopoulos and Badia, 2016) and the evaluation of newly emerging immersive virtual reality and augmented reality systems in their value for education and training. Further, by understanding and examining how eye tracking can be used to identify and predict learners’ interactions with computer-based systems, the methodologies and findings from this paper can further improve human-computer interaction literature. Eye-tracking methodologies used within this paper, such as the identification of actions and the order in which they occur, can be used to improve how studies validate their systems and identify human-computer interactions that can be scaffolded and improved. For example, eye movements can be used to detect cognitive load during programming tasks (Katona, 2022), identifying source code defects (Sharif et al., 2012), implementing scaffolds within immersive virtual reality environments (Bacca-Acosta et al., 2022).

## 10. Conclusion and future directions

The goal of this study was to examine, using multimodal data, how restricting agency during game-based learning supports learners SRL SMART operations and how the temporal deployment of these operations relate to learning outcomes. This paper established that it is important to consider how learners sequentially transition across these operations and how scaffolds within GBLEs can be used to support the adequate use of SRL SMART operations. From the findings of this study, we conclude that restricted agency is a sufficient scaffold of SMART operations in which learners who were scaffolded demonstrated increased learning outcomes and adequate deployment of SMART operations compared to learners who were not scaffolded during game-based learning. As such, this study expands the field of SRL in suggesting a temporal relationship between SMART operations and carving a path for future research in understanding how scaffolds should be implemented within GBLEs to support learners’ accurate and efficient use of SRL SMART operations. Future directions should aim to understand how SMART operations are deployed as time progresses, not just in relation to the previous operation that was deployed. Further, more studies are needed to further understand the following questions: Why are some transitions between SMART operations detrimental to learning outcomes? How can adaptive scaffolding support learners’ developing expertise of SRL SMART operation use? Are the results of this study generalizable to other GBLEs and learning technologies (e.g., intelligent tutoring systems, simulations, immersive environments)? How can other multimodal data unveil how SMART operations are operationalized and captured during game-based learning?

## Data availability statement

The datasets for this article are not publicly available due to IRB restrictions stating that all collaborators who request access to de-identified data need to be approved by the Principal Investigator. Requests to access the datasets should be directed to the corresponding author.

## Ethics statement

The studies involving humans were approved by North Carolina State University Institutional Review Board. The studies were conducted in accordance with the local legislation and institutional requirements. The participants provided their written informed consent to participate in this study. The individual(s) provided their written informed consent for the publication of any identifiable images or data presented in this article.

## Author contributions

DD contributed to the conceptualization, formal analysis, writing of the original draft, and visualization curation. MW contributed to the conceptualization, formal analysis, and writing of the original draft. SR contributed to the writing of the original draft and review and editing. KS and MP contributed to the writing of the original draft and visualization curation. NS contributed to the conceptualization and writing of the original draft. JL contributed to the review and editing of the draft, funding acquisition, and software. RA contributed to the conceptualization, funding acquisition, supervision, and review and editing of the original draft.
